# A Systems Biology Approach to Drug Targets in *Pseudomonas aeruginosa* Biofilm

**DOI:** 10.1371/journal.pone.0034337

**Published:** 2012-04-16

**Authors:** Gunnar Sigurdsson, Ronan M. T. Fleming, Almut Heinken, Ines Thiele

**Affiliations:** 1 Center for Systems Biology, University of Iceland, Reykajavík, Iceland; 2 Faculty of Industrial Engineering, Mechanical Engineering and Computer Science, University of Iceland, Reykajavík, Iceland; Virginia Commonwealth University, United States of America

## Abstract

Antibiotic resistance is an increasing problem in the health care system and we are in a constant race with evolving bacteria. Biofilm-associated growth is thought to play a key role in bacterial adaptability and antibiotic resistance. We employed a systems biology approach to identify candidate drug targets for biofilm-associated bacteria by imitating specific microenvironments found in microbial communities associated with biofilm formation. A previously reconstructed metabolic model of *Pseudomonas aeruginosa* (PA) was used to study the effect of gene deletion on bacterial growth in planktonic and biofilm-like environmental conditions. A set of 26 genes essential in both conditions was identified. Moreover, these genes have no homology with any human gene. While none of these genes were essential in only one of the conditions, we found condition-dependent genes, which could be used to slow growth specifically in biofilm-associated PA. Furthermore, we performed a double gene deletion study and obtained 17 combinations consisting of 21 different genes, which were conditionally essential. While most of the difference in double essential gene sets could be explained by different medium composition found in biofilm-like and planktonic conditions, we observed a clear effect of changes in oxygen availability on the growth performance. Eight gene pairs were found to be synthetic lethal in oxygen-limited conditions. These gene sets may serve as novel metabolic drug targets to combat particularly biofilm-associated PA. Taken together, this study demonstrates that metabolic modeling of human pathogens can be used to identify oxygen-sensitive drug targets and thus, that this systems biology approach represents a powerful tool to identify novel candidate antibiotic targets.

## Introduction

*Pseudomonas aeruginosa* (PA) is an opportunistic human pathogen and has an extremely versatile lifestyle. PA infection is life-threatening for immuno-compromised patients and PA is a feared pathogen for burn centers as well as a leading cause of death in cystic fibrosis (CF) patients [1], [2], [3]. PA has a large genome with 6.3 million base pairs, which encodes for 5570 genes and is rich in quorum sensing molecules and virulence factors [3]. PA has the ability to form a biofilm, which is thought to contribute to its adaptability and versatile lifestyle [4]. Furthermore, PA is often resistant to treatment and develops antibiotic resistance over time [5].

**Figure 1:**
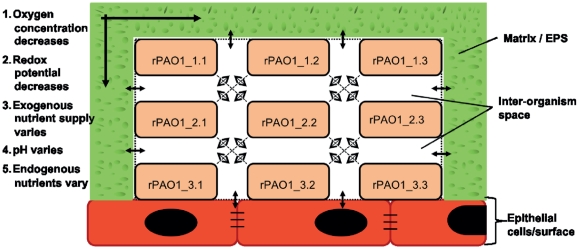
Schematic representation of a biofilm. This figure shows a schematic alignment of a biofilm and how nutrition and oxygen varies depending on the position of *P. aeruginosa* (PAO1) within the biofilm. From top to bottom and from the edge to the middle, the oxygen concentration decreases. The nutrient supply is more limited at the bottom and in the middle than on the top and at the boundaries of the biofilm. The redox potential also decreases from top to bottom. The pH value and endogenous nutrient concentrations thus vary according to the position of PA in the biofilm. The colonies PAO1_1.1 and PAO1_1.3 have the highest oxygen and nutrient supply, while for PAO1_3.2, these factors are most limited.

Biofilm is a surface attached, matrix enclosed microbial community, which is thought to play an important role in bacterial robustness. Biofilm-associated infections are more persistent than planktonic infections and more resistant to antibiotic treatment [6], [7]. There are three hypotheses why this is the case. First, the extracellular polymeric substance (EPS) secreted by bacteria in their surrounding leads to slower penetrations of antibacterial compounds. Second, few resistant genotypes may be in the colony and spread the resistance throughout the biofilm and thus, maintain the infection. Third, the idea of a dormant zone existing at the basal layer of the biofilm has been formulated [8]. This zone has little metabolic activity because of low nutrient supply and thus, is “dormant”. However, most antibiotics are dependent on bacterial replication and metabolic activity and therefore, bacteria located in this zone may survive antibiotic treatment. It has also been shown that hypoxia contributes to antibiotic resistance in PA biofilm [9]. Figure 1 shows a schematic drawing of a biofilm and the nutrient supply across the biofilm. In general, a biofilm infection is thought to be particularly important in certain clinical settings, such as prosthetic infections [7], in CF [10], [11], burn patients [12], and in endocarditis [8].

Biomedical questions, such as finding new drug targets, looking for better or novel biomarkers [13], and studying the metabolism of human diseases [14], can be addressed using systems biology [15]. Computational models are at the core of this effort. Genome-scale metabolic reconstructions are genomically, genetically and biochemically structured knowledge-bases [16]. There are metabolic reconstructions for an increasing number of organisms, including many bacteria, such as the human pathogens *Helicobacter pylori* [17], *Staphylococcus aureus* [18], and *Mycobacterium tuberculosis* [19], *archaea* [20], [21] and eukaryotes, including *Homo sapiens* [22] and *Mus musculus* [23], [24]. These metabolic reconstructions can be converted into computational models in order to address biotechnological and biomedical questions using different approaches, including constraint-based modeling [25]. The basis of constraint-based reconstruction and analysis (COBRA) has been described elsewhere [26], [27]. COBRA has allowed the analysis of genome-scale biochemical networks in an organized and predictive manner with excessive reliance on unknown model parameters [27]. Condition-specific, reconstruction-derived models can be used to predict phenotypic behavior, such as growth on different substrates and the effect of gene deletion on growth rate (reviewed in [15], [28]).

In this study, we employed the published reconstruction of *Pseudomonas aeruginosa,* iMO1056 [29], which accounts for 1056 genes (19% of the genome), 893 metabolites, and 883 reactions (1092 including exchange reactions). It captures most of PA's known metabolic capabilities, such as the synthesis of amino acids, lipids, virulence factors, and the cell wall. iMO1056 was assembled in analogy to an established reconstruction process [30].

New antibiotics are not only urgently needed for PA, but for many major human pathogens. Metabolic drug targets are interesting candidates for antibiotics as they offer a broad spectrum of possibilities and are likely to be phylogenetically conserved [18]. It is important that drug targets are essential for the organism and not present in the host. In this study, we wanted to test whether PA's metabolic model (iMO1056) could be used to suggest novel drug targets. At the same time, we were interested in modeling some properties of the biofilm and to propose candidate drugs particularly effective against biofilm-associated bacteria. Generally, it would be interesting to see whether one could use a system biology approach to design strain-dependent antibiotics. Antibiotics, which are available today (e.g., β-lactams, tetracycline's, and cephalosporin), act on a group of bacteria, e.g., gram-negative, gram-positive, aerobes, or anaerobes. By developing strain-dependent antibiotics, one could avoid complications arising from eliminating also the commensal flora. Such medication could also lead to decreased use of broad-spectrum antibiotics, reducing the selective pressure for widespread antibiotic resistance. It has been suggested that multi-target therapeutics could be effective for the same reason [31], [32], which motivated us to perform a computational double deletion to extend the list of suitable drug-targets.

## Materials and Methods

### Metabolic reconstruction of PA

All simulations were done using a previously published genome-scale metabolic reconstruction of PA, iMO1056 [29]. We downloaded the reconstruction from the Papin laboratory website (http://bme.virginia.edu/csbl/downloads-pseudomonas.php). To improve the model's consistency, we modified it as follows. First, we removed all duplicate reactions, which had a reversible and an irreversible version by keeping the reversible reaction (SI material, Table S1). Subsequently, we refined the directionality of all network reactions using thermodynamic analysis [33], [34]. Reaction bounds were only changed when the predicted *in vivo* standard transformed Gibbs energy indicated a qualitatively reversible reaction to be irreversible. The purpose of this valuation was to remove thermodynamically infeasible loops [35]. Details on the changed reaction directionality can be found in Table S1. Using the resulting PA model, we calculated the essential genes for all simulation conditions provided by Oberhardt *et al*. [29], i.e., we set the bounds of all reactions associated with a particular gene to zero and optimized the knock-out model for biomass production. The results were compared with the ones reported by Oberhardt *et al*. [29].

### Simulation constraints

Using flux balance analysis (FBA) [27], we simulated two different medium conditions: rich medium (Lucia-broth) and minimal medium (M9 medium supplemented with glucose) as described in Oberhardt *et al*. [29]. We distinguished high oxygen conditions (high O_2_, v_O2_lb_  = −20 mmol.g_DW_^−1^. hr^1^, v_O2_ub_ = 0 mmol.g_DW_^−1^.hr^−1^) and microaerobic conditions (low O_2_, v_O2_lb_  = −5 mmol.g_DW_^−1^.hr^-1^, v_O2_ub_ =  0 mmol.g_DW_^−1^.hr^−1^), as reported in Thiele *et al*. [17]. Taken together, we simulated the four following conditions: rich medium – high O_2_, rich medium – low O_2_, minimal medium – high O_2_, and minimal medium – low O_2_. All constraints are listed in Table S2.

### Single gene deletion study

The effect of a single gene knock-out on the production of individual biomass precursors was determined by adding a demand reaction for each biomass precursor contained in the biomass reaction in the form of ‘DM_biomass_precursor’. The lower bound (lb) was set to 0mmol.g_DW_^−1^.hr^−1^ and the upper bound (ub) to 1000mmol.g_DW_^−1^.hr^−1^ for each demand reaction. Then, we deleted each gene in the model by setting the lower and upper bound of the associated reaction(s) to 0 mmol.g_DW_^−1^.hr^−1^ and maximized each biomass precursor separately. The obtained value was stored. This procedure was repeated for all simulation conditions. This analysis was repeated for all 1056 genes included in the PA reconstruction. Subsequent analysis considered only those genes that fit the following criteria i) abolished *in silico* growth (*i.e*., growth rate was 0 hr^−1^), ii) reported to be essential experimentally, and iii) reported to have no homologous genes in humans (based on Perumal *et al*. [36]). These criteria narrowed our focus to a set of 398 genes.

### Candidate drug targets from the single deletion study

To identify potential drug targets, we compared the prediction of essential genes in rich medium with experimental data [37], [38]. We then determined whether these *in silico* and *in vivo* essential genes had known homologous genes in the human genome (based on Perumal *et al*. [36]) as it was desirable that candidate drug targets are unique to PA. We extended this list to include genes that were found to be essential in minimal medium, but for which no experimental data were available. We also compared our drug targets with the Drugbank database (http://www.drugbank.ca/) [39] to ensure that none of these predictions were already targeted.

### Double gene deletion study

To identify synthetic lethal genes, which could serve as candidate drug targets, we systematically deleted all combinations of the non-essential and non-homologue genes, and determined the *in silico* growth rate of the double knockout mutants by maximizing for biomass production. We repeated this procedure for all simulation conditions.

### Software

All computational simulations with iMO1056 were performed using MATLAB (The MathWorks Inc.). GLPK (GNU Linear Programming Kit) was used as a linear programming solver. The constraint-based methods for all simulations were performed in MATLAB using the COBRA toolbox [40], [41].

## Results

In this study, we aimed to identify candidate drug targets for biofilm-associated bacteria using a systems biology approach. We employed a previously published genome-scale metabolic reconstruction of *Pseudomonas aeruginosa* (PA), iMO1056, to compute single and double gene essentiality in different microenvironments typically found in microbial communities associated with biofilm. The results were analyzed with specific emphasis on oxygen-sensitive mutants. The list of candidate metabolic drug targets was reduced to only include targets without human homologues.

### Simulating different environments

To study the effect of different growth environments typical for planktonic and for biofilm-associated PA, we made the following assumptions. The planktonic PA encounters generally nutrient-rich and high oxygen conditions. In contrast, biofilm-associated PAs have different microenvironments that span nutrient rich to nutrient-poor and oxygen-rich to oxygen poor conditions (Figure 1). We simulated these four different environmental conditions, where nutrient rich, high oxygen corresponds to a planktonic microenvironment and nutrient poor, low and high oxygen, and nutrient rich, low oxygen to biofilm microenvironments. We wanted to investigate pathways and genes that were only used in biofilm growing bacteria but not in planktonic bacteria. Therefore, we computed the corresponding growth phenotype for wildtype PA and single knockout PA. As expected, different essential genes were observed for rich and minimal medium but no additional essential genes could be observed when changing from high to low oxygen uptake (data not shown).

### Candidate drug targets from the single deletion study

Based on our criteria for drug candidates, described in the method section, we identified 41 candidate metabolic drug targets in iMO1056. A total of 16 of these potential drug targets belonged to the lipid and cell wall synthesis subsystems and are specific for PA. Further 19 essential genes belonged to the amino acid metabolism subsystem. These genes represent ideal drug targets since they are not homologous to any human genes because they are all a part of the indispensable amino acids in man. Finally, there were six essential genes associated with cofactor synthesis subsystem.

In rich medium, we identified 21 as non-essential *in silico*, but which are known to be essential *in vivo* (false-negatives, Table 1, Table S3). These genes have no homologous genes in the human genome and could therefore also be potential drug targets. Many of these genes affected the same subsystems as listed above; however, some of the essential genes affected central metabolic functions, such as the TCA cycle (PA1582, Sdh) and Glycolysis/Gluconeogenesis (PA4043, Fba) (Table 1). In contrast, in rich medium, 29 of 54 genes with no human homolog were non-essential *in vivo* and can thus be considered as false-positives. In minimal medium, the number of false-positives was 48 of 74 genes.

**Table 1:** This table lists all genes, which abolished PA growth in a single gene deletion and which had no homologous genes in the human genome.

Subsystem	*In silico* + experimental evidence	Experimental evidence	*In silico* evidence	*In silico* minimal medium
Alanine biosynthesis			Alr (PA4930)	
Arginine, putrescine, and spermidine metabolism		UreB (PA4867)		ArgB (PA5323)
Aromatic amino acids	AroC (PA1681), AroK (PA5039)		AroB (PA5038), AroA (PA3164), AroE (PA0025)	TrpA (PA0035), TrpB (PA0036), PheA (PA3166)
Biosynthesis of cofactors, prosthetic groups and carriers, cell wall/ Lipopolysaccharide/ capsule		UppS (PA3652)		
Biotin biosynthesis	AccC (PA4848)			
Branched chain amino acid biosynthesis				IlvC (PA4694), IlvD (PA0353)
Cell envelope biosynthesis	GlmU (PA5552), GlmS (PA5549), MurI (PA4662), MraY (PA4415), MurE (PA4417), MurC (PA4411), MurB (PA2977)		DdlB (PA4410), DdlA (PA4201), MurA (PA4450),MurD (PA4414)	
Cell envelope biosynthesis- O-antigen	RmlA (PA5163)		RmlC (PA5164) RmlB (PA5161)	
Citrate acid cycle		SdhC (PA1581), SdhD (PA1582)		
Coenzyme A biosynthesis	CoaE (PA4529), Dfp (PA4848)		PanE (PA4397) PanB (PA4729) PanC (PA4730)	
Cysteine metabolism			CysC (PA1393) CysH (PA1756)	
Fatty acid biosynthesis			FabB (PA1609)	
Folate metabolism	FolB (PA0582), FolP (PA4750)	FolK (PA4728)	PhoA (PA3296)	
Fructose and mannose metabolism		MtlZ (PA2344)		
Glutamate and glutamine Biosynthesis		GlnA (PA5119)		
Glycine, serine, and threonine metabolism, aminoacyl-tRNA biosynthesis		GlyQ (PA0009), GlyS (PA0008)		
Glycolysis/ gluconeogenesis		Fba (PA0555)		
Histidine metabolism	HisE (PA5067)			HisG (PA4449), HisD (PA4448), HisB (PA5143), HisI (PA5066), HisA (PA5141)
Isoprenoid biosynthesis		IspA (PA4043)		
Lipopolysaccharide biosynthesis, core			RfaD (PA3337)	
Lipopolysaccharide biosynthesis, lipid A	KdsB (PA2979,) LpxB (PA3643), LpxK (PA2981), LpxA (PA3644)		Kds (PA4458)	
Membrane lipid metabolism	AccA (PA3639), AccB (PA4847), AccD (PA3112) Psd (PA4957)		PssA (PA4693)	
Methionine metabolism				MetX (PA0390)
NAD biosynthesis			NadD (PA4006)	
Oxidative phosphorylation		SdhA (PA1583), SdhB (PA1584), LldA (PA2382)		
Peptidoglycan biosynthesis			BacA (PA1959)	
Phenylalanine, tyrosine and tryptophan biosynthesis				TrpF (PA3113)
Purine and pyrimidine biosynthesis		PyrF (PA2876)	PurH (PA4854), PurB (PA2629), PurF (PA3108), PurM (PA0945), PurC (PA1013)	
Pyridoxine metabolism				ThrC (PA3735)
Riboflavin metabolism	RibD (PA4056)	RibA (PA4047)	RibB (PA4054)	
Tetrapyrrole biosynthesis		CobA (PA1778)		
Thiamine metabolism		ThiL (PA4051)		
Threonine and lysine metabolism	LysC (PA0904)	Asd (PA3117)		Hom (PA3736)
Ubiquinone biosynthesis		UbiG (PA3171)		
Vitamin B6 metabolism		PdxA (PA0593)		

These genes were sorted based on available evidence for their *in vivo* essentiality in literature [37], [38]. The first row represents genes that were essential *in silico* and have experimental data showing their essentiality. The second column lists genes that were reported to be essential experimentally but were not predicted to be essential in the *in silico* analysis. The genes in last two columns had no experimental support but were found to be essential *in silico*.

### Oxygen dependent use of genes and pathways in PA

By comparing the different effects of single gene deletion on the biomass precursor synthesis between normal and low O_2_ level, we could identify reactions affecting the metabolism singularly due to the oxygen level. We found 63 genes that had different effects on the biomass precursor synthesis between normoxic rich medium and hypoxic rich medium conditions. Of these 63 genes, 40 concerned the amino acid metabolism, 14 were in central metabolism, and nine genes belonged to other subsystems. For the two minimal medium growth conditions, we found 29 genes, which showed different growth phenotypes when deleted. Ten of them concerned the amino acid metabolism, 11 were in central metabolism, and eight genes belonged to other subsystems. Due to the difference in medium composition of rich and minimal medium, there were fewer essential genes in rich medium and subsequently, more oxygen-sensitive genes in rich medium. This oxygen-dependency was not observed in minimal medium due to growth essentiality of these genes. Taken together, we could identify a core set of 28 oxygen-dependent genes in PA (Table 2).

**Table 2:** Oxygen-sensitive genes**.**

Subsystem	Genes
Amino acid catabolism	FolD (PA1796), FdnI (P4810), FdnH (PA4811), FdnG (PA4812)
Amino acid synthesis	LysC (PA0904), Hom (PA3736), Ldh (PA3418), AruH (PA4976), GdhA (PA4588)
Central metabolism	Fda (PA0555), SdhC (PA1581), SdhD (PA1582), SdhA (PA1583), SdhB (PA1584), Fbp (PA5110), TktA (PA0548), AceA (PA2634), Edd (PA3194), GapA (PA3195)
Energy metabolism	PetC(PA4429), Ppa (PA4031)
Ethanol/ pyruvate metabolism	Pta (PA0835)
Nucleotide synthesis	Adk (PA3686), PyrB (PA0402), PyrC (PA3527), PyrF (PA2876)
Unassigned	CynT (PA2053)
Vitamin and cofactor synthesis	ThrC (PA3735)

This table lists all genes that were found to be oxygen-dependent in minimal and rich media conditions.

### Effect of single gene deletions on biomass precursor synthesis capability

**Figure 2:**
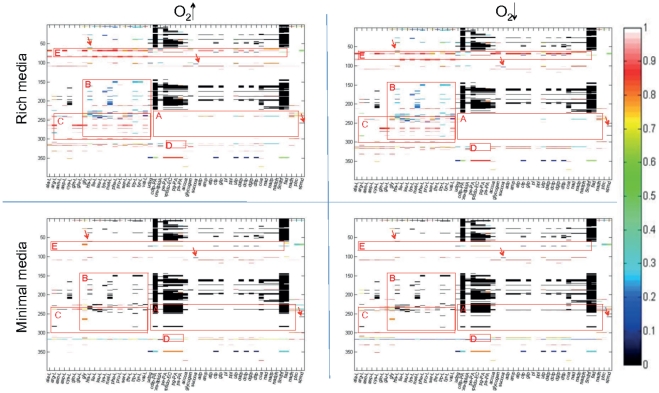
Essential genes for biomass precursor synthesis. This figure shows the results of the single gene deletion study, where the 398 candidate drug target genes are listed along the y-axis and the biomass precursors found in the biomass reaction [29] are listed on the x-axis. The color scale shows biomass precursor synthesis ability with zero corresponding to a complete loss of the precursor synthesis capability. Interesting areas in the four plots are highlighted with red boxes and discussed further in the text. *Box A* shows genes, which were essential in minimal but not in rich media. *Box B* corresponds to the area of amino acid precursors. We can see that in rich media the gene deletion had an effect on amino acid precursors but was not essential compared to minimal media, in which the gene deletion was essential. *Box C* highlights genes with subsystem effect. *Box D* shows those genes, which have a global effect but differed between minimal and rich media conditions. *Box E* shows similar tendency but a global effect is only observed in rich media conditions while gene deletion had a moderate effect in minimal media conditions. The arrows point to i) some few examples of single effect of gene deletion or ii) genes, whose deletion affected only a single precursor.

To refine our analysis, we calculated the effect of single gene deletion on the synthesis of the different biomass precursors (Figure 2). Apart from obvious differences in gene essentiality due to the lack of amino acids in the minimal medium, a comparison of the different growth conditions only revealed subtle differences between high and low O_2_ (Figure 2, Box A). Many of the essential genes in minimal medium caused a reduced biomass precursor synthesis rate in rich medium when deleted (Figure 2, Box B). We distinguished the following three categories depending on the effect of the gene deletion on different biomass precursors:

#### Case 1- local effect

27 genes affected the production of only single biomass precursors. For example, the deletion of PA5312 (KauB) affected only the synthesis of spermidine, PA5067 (HisE) only affected histidine synthesis, and PA1588 (SucC) only affected succinyl-coenzyme A synthesis under all environmental conditions (Figure 2, arrows).

#### Case 2- subsystem effect

The deletion of many genes affected the production of multiple biomass precursors, most of which belonged to the same category of precursors (e.g., amino acids and nucleotides). For example, the deletion of any of the genes in Box C (Figure 2) affected the production of amino acid precursors, which was the case for all environmental conditions. This result shows that the gene products synthesizing amino acids are acting very locally in the metabolic network.

#### Case 3 - global effect

There were seven genes, whose deletion had a global effect on the metabolic network. They affected the synthesis of 20 or more biomass precursors. For example, the deletion of PA3686 (Adk) affected the synthesis of 30 precursors under all environmental conditions. PA2634 (Icl_h) deletion had a global effect in rich media but had very little to no effect in minimal media. PA3194 (Edd) deletion had a global effect under all conditions but the set of precursors and the range of effects differed between environmental conditions. For example, the lipid precursors (Figure 2, Box D) were only affected in minimal media, regardless of the oxygen conditions. In contrast, deletion of PA0555 (Fba) had a global effect in rich media but almost no effect in minimal media. These globally acting genes are particularly interesting as drug targets since resistance may be hampered by their broad effect on metabolism.

### Double gene deletion study

**Figure 3:**
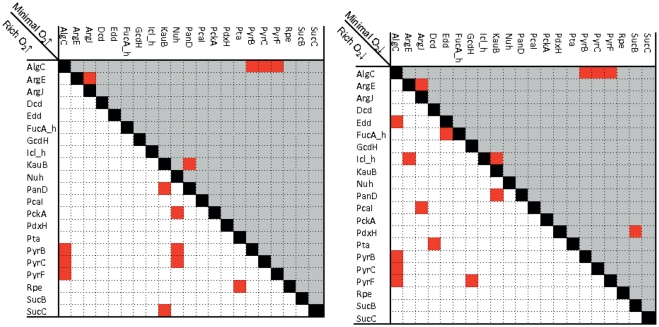
Synthetic essential genes. This figure shows the results of the double gene deletion study. White background corresponds to rich media and gray to minimal media. The diagram in the left hand side compare oxygen rich and the right hand side compares oxygen poor conditions. Red cells indicate essential double knockout combinations.

Double deletion study was performed for the 230 metabolic genes that were found to be non-essential in the single gene deletion analysis and for which no homologous genes could be identified in the human genome. We obtained 17 double knockout combinations, which could abolish iMO1056's growth, consisting of 21 distinct genes. Interestingly, we found different sets of combinations of essential double knockouts between the four environmental conditions (Figure 3). To understand why these combinations abolished iMO1056 growth and why this difference occurred between the conditions, we investigated the metabolic network topology. However, visual based analysis (based on iMO1056's metabolic map [29]) of the double knockout pairs turned out to be difficult for identification of a direct link between the individual pairs. While most reactions had neither clear visual links nor obvious metabolic clues in terms of reaction content, we could determine some metabolic relationships. For instance, PA5312 (KauB) and PA4731 (PanD) act both on beta-alanine (ala-B) metabolism. This metabolite was not present in the medium and only one other intracellular reaction (encoded by PA0132 (OapT)) was present in the reconstruction that was involved in ala-B metabolism. Our results suggest that the presence of only one pathway available was insufficient to sustain growth under oxygen-limited conditions and thus, the observed essentiality of the two genes could be explained. OapT was not considered in the double deletion study because it did not pass the criteria for a suitable drug target (see method section). The essentiality of the double knockout mutant PA5206 (ArgE) and PA4402 (ArgJ) can be explained by the fact that the reactions catalyzed by the corresponding gene products were a part of the same reaction chain. The deletion of these reactions abolished the flux through almost all possible reactions in the glutamine and glutamate synthesis, and thus in minimal medium, when these metabolites were not provided, the reactions associated to the deleted genes became essential.

## Discussion

The overall aim of this study was to determine metabolic phenotypic differences between *Pseudomonas aeruginosa* (PA) under different environmental conditions. In particular, we were interested in identifying candidate metabolic drug targets that reduce or abolish growth in biofilm-associated PA. Current antibiotics are most effective against replicating, metabolically active bacteria. In contrast, the dormant zone in a biofilm is insensitive towards this antibiotic treatment as bacteria located in this zone are mostly metabolically inactive. As metabolic drug targets do not depend on replication, they have the promise to selectively target slow growing bacteria found in biofilm. We performed systematic *in silico* single gene and double gene deletion studies to identify candidate metabolic drug targets potent to inhibit growth of biofilm-associated PA, in particular, bacteria growing in the lowest layer of the biofilm under hypoxic and nutrient poor conditions.

In the single gene deletion study, we evaluated the effect of each gene deletion on the synthesis of each precursor captured in the biomass reaction. This was done to increase the resolution of the single gene deletion. We were hoping to find genes that affected different precursor synthesis in the four different environments, especially, between the fast growing planktonic bacteria and the dormant zone of the biofilm. One may expect that slow growing bacteria in the dormant zone would maintain vital cellular functions, while omitting costly biosynthetic processes. We detected a few soft phenotypes with condition specific effect in hypoxic or nutrient poor media but, in general, we did not observe much growth difference of single deletion mutants between the simulated growth conditions. In fact, no single knockout disrupted growth singularly under hypoxic conditions. The single gene deletion analysis identified 41 potential drug targets for all four conditions. Moreover, 26 of the potential drug targets have been shown to abolish growth *in vivo* [37], [38] and thus, confirmed our *in silico* predictions under rich medium conditions. The remaining 15 drug targets were predict to be essential in minimal media and therefore may be of special interest for biofilm-associated bacteria.

Recently, Pommerenke *et al.* performed a genome-wide transposon mutant study of the *P. aeruginosa* strain A14 (4030 mutants in total) [42]. They determined a network of phenotypic relatedness between these mutants. Six genes predicted to be essential in our single gene deletion study (AccC, GlmU, Alr, AroB, NadD, and RibB) and three genes that were essential in our double knockout study (Edd, ArgJ, and SucC) were included in the global phenotypic network. The neighbors of these genes in the phenotypic landscape may be useful targets for identifying further candidate drug targets experimentally. Furthermore, Müsken *et al.* screened a set of transposon mutants for their ability to form biofilm [43]. They also identified genetic determinants promoting survival under microaerophilic conditions, which were essential for *P. aeruginosa* biofilm establishment. In total, 394 mutants were deficient in biofilm formation. Genes involved in arginine metabolism were particularly affected indicating that a functional arginine metabolism is essential for biofilm formation. Several genes involved in arginine biosynthesis as well as arginine and ornithine degradation caused reduced biofilm formation in the respective mutants. Although the genes identified in this mutant study were not found to be essential in our *in silico* analysis, we predicted another gene involved in arginine metabolism (ArgB) to be essential on minimal medium. Furthermore, we predicted a double knockout mutant of ArgE and ArgJ, whose gene products are involved in ornithine and arginine biosynthesis, to be nonviable on minimal medium (Figure 3). Thus, our *in silico* results also suggest an importance of arginine metabolism for biofilm formation and underline the value of a systems biology approach to identify candidate drug targets.

Ten genes, predicted to be essential in our single gene deletion study, also caused poor biofilm formation *in vivo*, when deleted [43]. The reason for the low number of matches is most probably that Müsken *et al.* targeted a different objective: the ability of PA to form biofilm rather than viability, although these two factors are probably correlated. Furthermore, the analysis was performed for the strain PA14 instead of PAO1. Six among the ten overlapping genes (AroB, AroC, PanB, PanC, IlvC, and TrpD), belonging to the subsystems of aromatic amino acid metabolism, branched chain amino acid biosynthesis, and coenzyme A biosynthesis, have no homolog in humans and are thus potential drug targets. The experimental observation that mutations in these genes cause deficiency in biofilm formation supports our *in silico* prediction that their gene products are indeed metabolic weak points of PA.

We performed a double gene deletion study to extend our candidate drug target list. Essential double knockout combinations are very difficult to predict solely based on reaction maps and biochemical knowledge; thus, they are ideal for a network-based analysis. Using the PA *in silico* model, we aimed to predict novel metabolic targets that could be followed up by experimental work. Considering the high number of possible double knockout mutants, computational approaches are needed to optimize experimental work and to increase the success rate, i.e., finding synthetic lethal mutants. The double deletion of non-essential genes with no homologous genes in the human genome resulted in a promising set of candidate drug targets. Two genes involved in pyrimidine metabolism (PyrB and PyrC) were predicted to be lethal in double knockouts involving AlgC (Figure 3). The deletion of both genes caused poor biofilm formation in the respective mutants *in vivo* [43] underlining that they may be indeed potential drug targets. Furthermore, we identified gene pairs predicted to be lethal in double deletion, which were hypoxic-sensitive (Figure 3). Pairing non-essential genes for multi-target drug medication could extend current treatment options greatly and may turn out to be more effective against PA than single drug target options. It has been also hypothesized that multi-targeting is less likely to induce antibiotic resistance [31]. As testing of all possible double knockout combinations may become experimentally intractable, the metabolic modeling of PA be a powerful approach to identify a testable subset. However, more systematic analysis and further studies combining experimental work and computational predictions will be necessary to refine the set of proposed candidate lethal double gene deletions under hypoxic conditions. If the numbers of possible pairs (52.900) and of *in silico* essential pairs (17) are considered, it is evident that the prediction of pairs to be investigated experimentally when relying solely on biochemistry and reaction maps would be extremely challenging.

The use of metabolic modeling for prediction of candidate drug targets is a growing research area in systems biology. For instance, iMO1056 has been used to propose 41 candidate drug targets for PA [44]. Furthermore, a metabolic network of *Burkholderia cenocepacia* has been reconstructed and drug targets have been proposed for this pathogen, which also infects cystic fibrosis patients [45]. Recently, Oberhardt *et al.* integrated microarray data from PA growing in the lung epithelium of a cystic fibrosis patient with iMO1056 [46]. The microarray data measured gene expression level at different time points to observe the adaptation of PA in a CF lung [46]. The authors also performed a single gene deletion study while simulating growth of PA under microaerobic conditions. The deletion of 54 genes with no homology to human genes has been predicted to be lethal on *in silico* synthetic cystic fibrosis medium [46]. Of those, 51 overlapped with genes predicted to be essential both on minimal medium and rich medium in our study. The three remaining genes (PyrB, PyrC, and PyrF) were predicted to be lethal in a double knockout with AlgC in our study. It is likely that these genes were nonlethal in our single gene deletion study due to differences in the simulated medium composition and constraints compared to the study setup by Oberhardt *et al*. Interestingly, the effect of gene deletion on growth rates changed when different isolate-specific models, representing PA infection at different time points, were simulated [46]. Their predictions of infection stage-specific drug targets, taken together with our predictions of drug targets specific for biofilm-grown PA, should be useful for designing drugs aiming for PA's metabolic weaknesses. It is expected that, as more data becomes available, the PA metabolic reconstruction will be improved further, which will ultimately improve the accuracy of the predictions. Experimental studies testing some of the predicted hypotheses will also lead to further refinement of the reconstruction content.

The presented work illustrates a systems biology approach to identify candidate metabolic drug targets that consider specific microenvironments found in biofilm. It shows that FBA can be used to determine key metabolic differences between planktonic and biofilm colonies and that this knowledge may be used to propose novel drug targets.

## Supporting Information

Table S1 **This table lists reactions that were modified in iMO1056.**
(XLSX)

Table S2 **This table lists the constraints implemented for the four simulated growth conditions.**
(XLSX)

Table S3 **This table lists genes that were essential in the silico single gene deletion study on rich medium and on minimal.** Genes that were experimentally demonstrated to be essential are shown for comparison. Furthermore, PA genes that have no human homolog are listed.
(XLSX)

## References

[1] Bodey GP, Bolivar R, Fainstein V, Jadeja L (1983). Infections caused by Pseudomonas aeruginosa.. Rev Infect Dis.

[2] Driscoll JA, Brody SL, Kollef MH (2007). The epidemiology, pathogenesis and treatment of Pseudomonas aeruginosa infections.. Drugs.

[3] Stover CK, Pham XQ, Erwin AL, Mizoguchi SD, Warrener P (2000). Complete genome sequence of Pseudomonas aeruginosa PAO1, an opportunistic pathogen.. Nature.

[4] Wang Y, Dai Y, Zhang Y, Hu Y, Yang B (2007). Effects of quorum sensing autoinducer degradation gene on virulence and biofilm formation of Pseudomonas aeruginosa.. Sci China C Life Sci.

[5] Carmeli Y, Troillet N, Eliopoulos GM, Samore MH (1999). Emergence of antibiotic-resistant Pseudomonas aeruginosa: comparison of risks associated with different antipseudomonal agents.. Antimicrob Agents Chemother.

[6] del Pozo JL, Patel R (2007). The challenge of treating biofilm-associated bacterial infections.. Clin Pharmacol Ther.

[7] Stewart PS, Costerton JW (2001). Antibiotic resistance of bacteria in biofilms.. Lancet.

[8] Hall-Stoodley L, Costerton JW, Stoodley P (2004). Bacterial biofilms: from the natural environment to infectious diseases.. Nat Rev Microbiol.

[9] Borriello G, Werner E, Roe F, Kim AM, Ehrlich GD (2004). Oxygen limitation contributes to antibiotic tolerance of Pseudomonas aeruginosa in biofilms.. Antimicrob Agents Chemother.

[10] Moreau-Marquis S, Stanton BA, O'Toole GA (2008). Pseudomonas aeruginosa biofilm formation in the cystic fibrosis airway.. Pulm Pharmacol Ther.

[11] Sriramulu DD, Lunsdorf H, Lam JS, Romling U (2005). Microcolony formation: a novel biofilm model of Pseudomonas aeruginosa for the cystic fibrosis lung.. J Med Microbiol.

[12] Bielecki P, Glik J, Kawecki M, Martins dos Santos VA (2008). Towards understanding Pseudomonas aeruginosa burn wound infections by profiling gene expression.. Biotechnol Lett.

[13] Kell DB (2006). Systems biology, metabolic modelling and metabolomics in drug discovery and development.. Drug Discovery Today.

[14] Obrzut S, Tiongson J, Jamshidi N, Phan HM, Hoh C (2010). Assessment of metabolic phenotypes in patients with non-ischemic dilated cardiomyopathy undergoing cardiac resynchronization therapy.. J Cardiovasc Transl Res.

[15] Oberhardt MA, Palsson BO, Papin JA (2009). Applications of genome-scale metabolic reconstructions.. Mol Syst Biol.

[16] Palsson B (2009). Metabolic systems biology.. FEBS Lett.

[17] Thiele I, Vo TD, Price ND, Palsson BO (2005). Expanded metabolic reconstruction of Helicobacter pylori (iIT341 GSM/GPR): an in silico genome-scale characterization of single- and double-deletion mutants.. J Bacteriol.

[18] Lee DS, Burd H, Liu J, Almaas E, Wiest O (2009). Comparative genome-scale metabolic reconstruction and flux balance analysis of multiple Staphylococcus aureus genomes identify novel antimicrobial drug targets.. J Bacteriol.

[19] Jamshidi N, Palsson BO (2007). Investigating the metabolic capabilities of Mycobacterium tuberculosis H37Rv using the in silico strain iNJ661 and proposing alternative drug targets.. BMC Syst Biol.

[20] Feist AM, Henry CS, Reed JL, Krummenacker M, Joyce AR (2007). A genome-scale metabolic reconstruction for Escherichia coli K-12 MG1655 that accounts for 1260 ORFs and thermodynamic information.. Mol Syst Biol.

[21] Gonzalez O, Gronau S, Falb M, Pfeiffer F, Mendoza E (2008). Reconstruction, modeling & analysis of Halobacterium salinarum R-1 metabolism.. Mol Biosyst.

[22] Duarte NC, Becker SA, Jamshidi N, Thiele I, Mo ML (2007). Global reconstruction of the human metabolic network based on genomic and bibliomic data.. Proc Natl Acad Sci U S A.

[23] Sigurdsson MI, Jamshidi N, Steingrimsson E, Thiele I, Palsson BO (2010). A detailed genome-wide reconstruction of mouse metabolism based on human Recon 1.. BMC Syst Biol.

[24] Selvarasu S, Karimi IA, Ghim GH, Lee DY (2010). Genome-scale modeling and in silico analysis of mouse cell metabolic network.. Mol Biosyst.

[25] Price ND, Reed JL, Palsson BO (2004). Genome-scale models of microbial cells: evaluating the consequences of constraints.. Nat Rev Microbiol.

[26] Palsson B (2006). Systems biology: properties of reconstructed networks. Cambridge: Cambridge University Press.. xii,.

[27] Orth JD, Thiele I, Palsson BO (2010). What is flux balance analysis?. Nat Biotechnol.

[28] Feist AM, Palsson BO (2008). The growing scope of applications of genome-scale metabolic reconstructions using *Escherichia coli*.. Nat Biotech.

[29] Oberhardt MA, Puchalka J, Fryer KE, Martins dos Santos VA, Papin JA (2008). Genome-scale metabolic network analysis of the opportunistic pathogen Pseudomonas aeruginosa PAO1.. J Bacteriol.

[30] Thiele I, Palsson BO (2010). A protocol for generating a high-quality genome-scale metabolic reconstruction.. Nat Protoc.

[31] Silver LL (2007). Multi-targeting by monotherapeutic antibacterials.. Nat Rev Drug Discov.

[32] Zimmermann GR, Lehar J, Keith CT (2007). Multi-target therapeutics: when the whole is greater than the sum of the parts.. Drug Discov Today.

[33] Fleming RM, Thiele I, Nasheuer HP (2009). Quantitative assignment of reaction directionality in constraint-based models of metabolism: application to Escherichia coli.. Biophys Chem.

[34] Fleming RM, Thiele I (2011). von Bertalanffy 1.0: a COBRA toolbox extension to thermodynamically constrain metabolic models.. Bioinformatics.

[35] Price ND, Thiele I, Palsson BO (2006). Candidate states of Helicobacter pylori's genome-scale metabolic network upon application of loop law thermodynamic constraints.. Biophys J.

[36] Perumal D, Lim CS, Sakharkar KR, Sakharkar MK (2007). Differential genome analyses of metabolic enzymes in Pseudomonas aeruginosa for drug target identification.. In Silico Biol.

[37] Jacobs MA, Alwood A, Thaipisuttikul I, Spencer D, Haugen E (2003). Comprehensive transposon mutant library of Pseudomonas aeruginosa.. Proceedings of the National Academy of Sciences.

[38] Lewenza S, Falsafi RK, Winsor G, Gooderham WJ, McPhee JB (2005). Construction of a mini-Tn5-luxCDABE mutant library in Pseudomonas aeruginosa PAO1: a tool for identifying differentially regulated genes.. Genome Res.

[39] Wishart DS, Knox C, Guo AC, Cheng D, Shrivastava S (2008). DrugBank: a knowledgebase for drugs, drug actions and drug targets.. Nucleic Acids Res.

[40] Schellenberger J, Que R, Fleming RM, Thiele I, Orth JD (2011). Quantitative prediction of cellular metabolism with constraint-based models: the COBRA Toolbox v2.0.. Nat Protoc.

[41] Becker SA, Feist AM, Mo ML, Hannum G, Palsson BO (2007). Quantitative prediction of cellular metabolism with constraint-based models: The COBRA Toolbox.. Nat Protocols.

[42] Pommerenke C, Musken M, Becker T, Dotsch A, Klawonn F (2010). Global genotype-phenotype correlations in Pseudomonas aeruginosa.. PLoS Pathog.

[43] Musken M, Di Fiore S, Dotsch A, Fischer R, Haussler S (2010). Genetic determinants of Pseudomonas aeruginosa biofilm establishment.. Microbiology.

[44] Perumal D, Samal A, Sakharkar KR, Sakharkar MK (2011). Targeting multiple targets in Pseudomonas aeruginosa PAO1 using flux balance analysis of a reconstructed genome-scale metabolic network.. J Drug Target.

[45] Fang K, Zhao H, Sun C, Lam CM, Chang S (2011). Exploring the metabolic network of the epidemic pathogen Burkholderia cenocepacia J2315 via genome-scale reconstruction.. BMC Syst Biol.

[46] Oberhardt MA, Goldberg JB, Hogardt M, Papin JA (2010). Metabolic network analysis of Pseudomonas aeruginosa during chronic cystic fibrosis lung infection.. J Bacteriol.

